# NTD Health: An electronic medical record system for neglected tropical diseases

**DOI:** 10.7705/biomedica.6269

**Published:** 2022-12-01

**Authors:** Rodrigo Ochoa, Alessa Álvarez, Jordan Freitas, Saptarshi Purkayastha, Iván D. Vélez

**Affiliations:** 1 Programa de Estudio y Control de Enfermedades Tropicales - PECET, Facultad de Medicina, Universidad de Antioquia, Medellín, Colombia Universidad de Antioquia Universidad de Antioquia Medellín Colombia; 2 Max Planck Tandem Group in Biophysics of Tropical Diseases, Universidad de Antioquia, Medellín, Colombia Universidad de Antioquia Universidad de Antioquia Medellín Colombia; 3 Centro para la Cuarta Revolución Industrial de Colombia, Complejo Ruta N, Medellín, Colombia Centro para la Cuarta Revolución Industrial de Colombia Medellín Colombia; 4 Department of Computer Science, Loyola Marymount University, Los Angeles, USA Loyola Marymount University Loyola Marymount University Los Angeles USA; 5 Department of BioHealth Informatics, School of Informatics and Computing, Indiana University- Purdue University Indianapolis, Indianapolis, USA Purdue University Purdue University Indianapolis Indianapolis USA

**Keywords:** Neglected tropical diseases, electronic health records, public health informatics, software, enfermedades desatendidas, registros electrónicos de salud, informática en salud pública, programas informáticos

## Abstract

**Introduction:**

The use of technological resources to support processes in health systems has generated robust, interoperable, and dynamic platforms. In the case of institutions working with neglected tropical diseases, there is a need for specific customizations of these diseases.

**Objectives:**

To establish a medical record platform specialized in neglected tropical diseases which could facilitate the analysis of treatment evolution in patients, as well as generate more accurate data about various clinical aspects.

**Materials and methods:**

A set of requirements to develop state of the art forms, concepts, and functionalities to include neglected tropical diseases were compiled. An OpenMRS distribution (version 2.3) was used as reference to build the platform, following the recommended guidelines and shared-community modules.

**Results:**

All the customized information was developed in a platform called NTD Health, which is web-based and can be upgraded and improved by users without technological barriers.

**Conclusions:**

The electronic medical record system can become a useful tool for other institutions to improve their health practices as well as the quality of life for neglected tropical disease patients, simplifying the customization of healthcare systems able to interoperate with other platforms.

## Resumen

**Introducción.:**

El uso de recursos tecnológicos destinados a apoyar procesos en los sistemas de salud ha generado plataformas sólidas, interoperables y dinámicas. En el caso de las instituciones que trabajan con enfermedades tropicales desatendidas, existe la necesidad de personalizaciones específicas en las herramientas de uso médico.

**Objetivos.:**

Establecer una plataforma para historias clínicas especializada en enfermedades tropicales desatendidas, con el fin de facilitar el análisis de la evolución del tratamiento de los pacientes, además de generar datos más precisos sobre diversos aspectos clínicos.

**Materiales y métodos.:**

Se compiló un conjunto de requisitos para implementar formularios, conceptos y funcionalidades que permitan incluir enfermedades tropicales desatendidas. Se utilizó una distribución de OpenMRS (versión 2.3) como referencia para construir la plataforma, siguiendo las pautas recomendadas y módulos compartidos por la comunidad.

**Resultados.:**

Toda la información personalizada se implementó en una plataforma llamada NTD Health, la cual se encuentra almacenada en la web y los usuarios pueden actualizarla y mejorarla sin barreras tecnológicas.

**Conclusiones.:**

El sistema de historias clínicas electrónicas puede convertirse en una herramienta útil para que otras instituciones mejoren sus prácticas en salud, así como la calidad de vida de los pacientes con enfermedades tropicales desatendidas, simplificando la personalización de los sistemas de salud capaces de interoperar con otras plataformas.

Neglected tropical diseases are a set of infectious diseases affecting mainly disadvantaged populations in tropical and subtropical areas of the world ^1^. The prevalence of neglected tropical diseases is determined by different factors, including environmental, social, and economic conditions, as well as cultural practices and sanitation infrastructure development ^2^. In addition, there is a lack of economic and financial resources intended for research on neglected tropical diseases, and low interest of the pharmaceutical industry in response to the economic features associated with the affected population ^3^. Also, there is an association between the prevalence of neglected tropical diseases and the low budget allocated to health care centers. This has resulted in multiple philanthropic agencies coming together to create organizations that could improve neglected tropical disease visibility ^4^^-^^6^.

Under these conditions, management of these diseases can be challenging, which is why different support tools have been developed, such as the electronic medical record systems. With several free and open-source systems, the transition of medical records from paper to digital format has been beneficial, especially in developing countries ^7^^,^^8^. The main advantages of these free and open-source electronic medical record systems are lower implementation costs, fewer restrictions around content personalization, more efficient information gathering and processing, and fewer code development efforts ^9^.

To select the most appropriate platform for a suitable workflow for electronic medical record systems, certain criteria are considered, such as the possibility of customizing the content without the need for extensive knowledge in computer programming. Likewise, an offline data registration is also important so that the process is independent of internet access, which is sometimes unavailable in underdeveloped areas ^10^^,^^11^.

Among the free and open source electronic medical record systems. OpenMRS has a modular system that can be adapted to the needs of various contexts, and is able to create metadata according to the needs of each institution (https://openmrs.org/) ^12^. It has a large dictionary of concepts with ease of creating new ones based on clinical needs, such as disease specific terms. In addition, OpenMRS allows personalizing the forms and reports according to the health issue and the needs of information analysis.

The implementation of an electronic medical record system allows the integration of other information platforms within the same medical center, and in the health system of territories ^13^^,^^14^. These other systems include laboratory test reports, management of clinical information for each patient, and smartphones applications that allow data collection in isolated areas of the territory. This integration allows interoperability for a correct data management that can be shared in a safe and reliable way ^15^.

The objective of this study was based on the implementation of a personalized electronic medical record system based on OpenMRS, in order to manage clinical information of patients with neglected tropical diseases. The system was adapted to the specific needs of the *Programa para el Control de Enfermedades Tropicales* (PECET) at the *Universidad de Antioquia*, Medellín, Colombia, using specialized forms and concepts associated with each disease.

## Materials and methods

### Compiling requirements

We compiled a set of requirements to supply state-of-the-art forms, concepts and functionalities related to a variety of neglected tropical diseases. Specifically, we adapted an OpenMRS distribution (reference application 2.3) to record data for diseases such as dengue, chikungunya, Zika and leishmaniasis. According to the current OpenMRS modules and functionalities, we focused the deployment on four categories: disease-specific forms, authentication and roles, diagnosis, and drug orders. The topics were categorized based on the time required for development and implementation, complexity of each task and availability of functions previously created for OpenMRS.

### Deployment of forms and concepts

Different disease-specific forms were built using the HTML Form Entry (HFE) module of OpenMRS. With the HFE module it is possible to develop forms using HTML, CSS, JavaScript, and new HTML5 features like offline storage and form validation, facilitating the entry through any web browser. Epidemiological and clinical data were collected through a set of questions and add-ons, e. g. human body schemes to report the location of leishmaniasis lesions. The electronic medical record system was adapted to the MCL/CIEL dictionary (http://www.maternalconceptlab.com) distributed along with some OpenMRS packages. In addition, a set of concepts not included in the original list were included, and consequently submitted to the concept project in order to be shared with the OpenMRS community. The concepts were mapped (if available) with other controlled medical vocabularies, such as SNOMED CT (http://www.snomed.org/snomed-ct) and ICD-10 (http://www.icd10data.com/). Finally, we added forms to each patient’s dashboard and managed the records based on defined roles.

To develop the platform infrastructure, the customized electronic medical record system was deployed on a server running Debian Linux on Apache Tomcat, v. 7 (http://tomcat.apache.org/), which is the recommended Java servlet container. Our electronic medical record system can also be deployed to other Java application servers, such as JBoss or WebSphere. The OpenMRS data model and the concept dictionary were managed in a MySQL database server, using English and Spanish as the main languages used for metadata. Web-based access was provided to the health care personnel from PECET, involved in the attention of patients affected by various tropical viral diseases and American cutaneous leishmaniasis. The forms for dengue, chikungunya and Zika virus were compacted into one, due to their similar symptoms, epidemiological conditions, and personal clinical histories.

### Dissemination and training

To promote the open-source technologies behind the electronic medical record system implementation, a local workshop called “Health Informatics Tools” was conducted to train users and developers from Colombia and other Latin American countries (http://ubmc-pecet.udea.edu.co/hitworkshop/). Different tools such as OpenMRS, DHIS2 (https://dhis2.org), and Sana (http://sana.mit.edu/) were included. Simultaneously, an open course was developed for OpenMRS users, implementers, and developers around the world. The course contains information reviewed by experts, with videos and various methodologies that can help guide the migration and implementation of platforms such as NTD Health in other scenarios.

**Figure 1:**
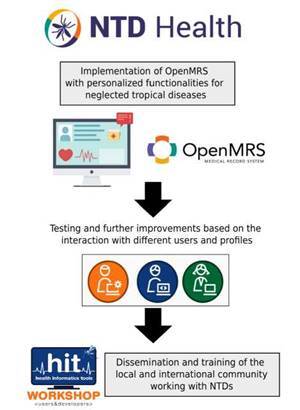
Neglected tropical disease health implementation strategy. The process included the implementation of a customized OpenMRS platform with self-generated metadata. The project also involves dissemination and training for the local and international community through events and online educational material.

The implementation strategy covered in NTD Health is summarized in figure 1.

### Ethical approval

This project did not involve the participation or experimentation with human or animal participants.

## Results

### Deployment strategy

The enterprise version of the OpenMRS reference application 2.3 was used for NTD Health. To personalize the system, the reference application and the UICommons modules were modified to include the NTD Health logo and other stylesheet changes in the source code. We used Spanish translation because the implementation was originally intended for Latin American institutions. At the time of the deployment, most of the terms were not translated appropriately due to the new release of the reference application. For this reason, we supported the translation of terms from multiple implemented modules. Specifically, the OrderEntryUI module was completely translated and made publicly available to the entire OpenMRS community.

**Figure 2:**
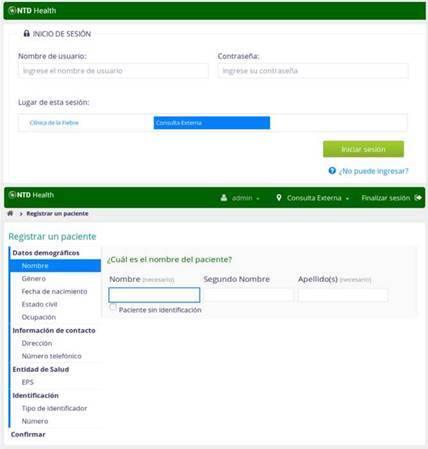
Screenshots of the login and registration forms from the NTD Health implementation of the *Programa de Estudio y Control de Enfermedades Tropicales* (PECET). The forms describe the specific data required in Spanish for the people affiliated with this electronic medical record platform.

To collect local and personalized patient data, the registration form app was modified from the source code and complemented with additional questions about country identification, occupation, and health institution where the patient is affiliated were included (figure 2). Details regarding the diseases were organized in forms, and further added to the system through the HTMLFormEntry module, following OpenMRS and general electronic medical record guidelines ^16^. Three forms were designed to obtain information about tropical viruses, personal history, and epidemiological background of the patients. All the information required was designed by health personnel involved in the management of these diseases, aiming at providing all the necessary data to improve the diagnosis and future treatments.

The created forms, together with the defined MCL/CIEL concepts are available in the GitHub repository: https://github.com/rochoa85/NTDHealth_Forms._  The use of this controlled vocabulary to add new medical concepts associated with neglected tropical diseases is crucial to allow the interoperability of the platform with other local electronic medical record systems, facilitating the potential communication between them. Data storage is managed through local platform installations, which means that nothing is stored in public servers, providing the security and confidentiality necessary to administer clinical information, also giving safe and long-term storing conditions. In addition, OpenMRS generates copies of the information at certain time intervals to avoid losing sensitive data.

**Figure 3:**
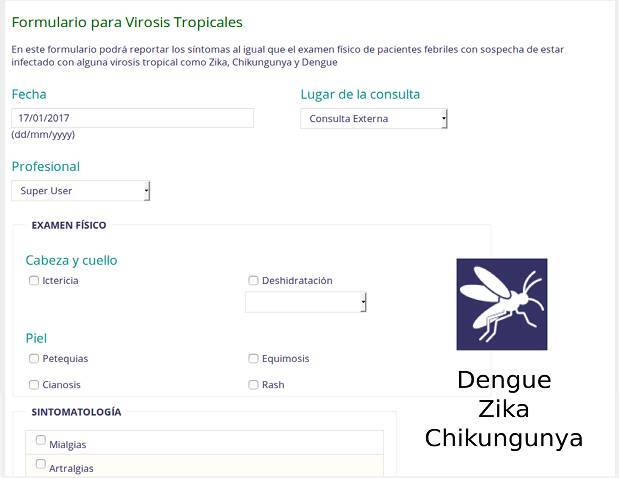
Screenshot of the tropical virus form for dengue, Zika and chikungunya. General information in Spanish with specific questions about physical examination, symptoms and diagnostics is included.

Regarding tropical viruses, we focused our attention to those transmitted by the same insect vector (*Aedes aegypti*), which show similar symptoms and are usually difficult to diagnose by health professionals from endemic territories ^17^. This set of infections include those of Zika, dengue and chikungunya, being all three characterized by general and specific disease- related data captured through physical examination, symptomatology, and diagnostic inferences (figure 3). To obtain a broader panorama of the diseases, data from personal history with epidemiological background was captured, including information about living conditions, traveling sites, duration of the travel, and potential exposure based on frequent activities.

**Figure 4:**
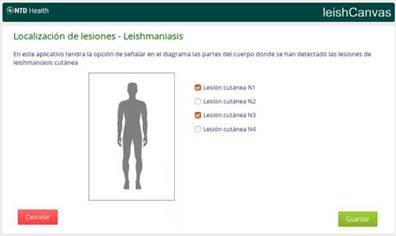
Screenshot of the OpenWebApp canvas for the localization of cutaneous leishmaniasis lesions in a human body scheme.

Finally, due to the expertise of the PECET research group in leishmaniasis, we used the OpenWebApp functionalities in OpenMRS to include a human body canvas on which the location of cutaneous lesions can be recorded graphically, along with other descriptions. A snapshot of the application is provided in figure 4.

The platform provides an open and useful way to document updated reports and improve the management of these diseases in isolated institutions which require automatized health information tools.

### Dissemination impact

**Figure 5:**
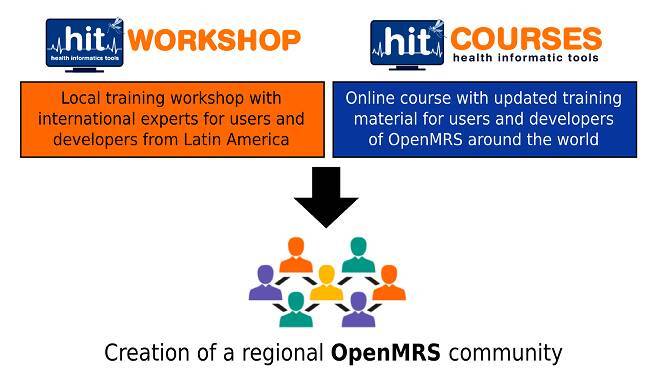
Scheme describing the local workshop and the online course for OpenMRS training. Both activities were aligned to improve the knowledge of health informatics tools in Latin America, looking to create a regional OpenMRS community.

Two strategies were carried out to reach personally and virtually a group of users and developers in Colombia and other countries around the world. A summary of the strategies is shown in figure 5.

In the local workshop, we had the participation of various attendees from different sectors including engineers, health professionals, students, and entrepreneurs. The topics were taught using different methodologies, such as conference sessions, hands-on training, lightning talks, and a hackathon related to the needs and possible solutions in health informatics tools for real problems, including the NTD Health platform ^18^.

The online course provides valuable and updated resources for people entering the world of OpenMRS, containing information about how to install the latest releases of OpenMRS under different environments, and more elaborated topics such as the customization and overriding of system variables, and manipulation of the data model. At the end, the goal is to provide sustainable and community-driven support to the project to facilitate the inclusion of novel modules and functionalities required by the NTD Health users in the future.

## Discussion

The implementation of electronic medical records has been useful to improve the efficiency of storing and analyzing clinical data, enabling meta-analysis and execution of data mining protocols to learning about epidemiological conditions, drug side effects and other evidence to improve disease diagnosis ^19^. Due to the lack of data associated with neglected tropical diseases, the use of electronic platforms to store and manage clinical information is particularly relevant in order to improve research, curate novel datasets, and provide better healthcare services to the affected populations ^20^^,^^21^.

After surveying some users of the platform, we noticed that most of the health professionals still use paper forms and excel files to store clinical data, although the process is commonly disrupted due to the rigid structure of the storage system and lack of efficient synchronization across the users. As a secondary finding of this work, it was clear that a multidisciplinary team is a key element for establishing efficient data collection techniques. The use of platforms by diverse groups of physicians, microbiologists, chemists, engineers, and nurses, facilitate their exchange of information and enhances the process of managing neglected tropical disease related research and patient care.

As perspectives, we highlight how cloud-based accessibility could be useful in zones where clinicians are normally collecting the data. This will allow them to store data in real time and synchronize it quickly with information stored by other professionals. In fact, the system can operate in surroundings with lower connectivity resources, given the light HTML content included in the interface that usually does not demand a high bandwidth size to be accessed. This is also supported by the option of installing NTD Health as a stand-alone application in the healthcare center.

Regarding the usability of the interface, the OpenMRS reference application provides friendly functionalities for people that are not familiar with this kind of technology, which is one of the main barriers for health professionals to adopt such platforms in their daily activities. The platform can be subsequently customized to add user-friendly HTML forms covering other tropical diseases, as well as to adapt the questionnaires and data acquisition pipelines based on the internal country regulations.

Overall, NTD Health is a customized electronic medical record system based on OpenMRS that provides forms and concepts required for the clinical care of patients diagnosed with tropical diseases. The collaborative and open nature of the project allows us to share the information with the community, and consequently incorporate their feedback and contributions to improve the system. The project is supported by a set of novel forms and concepts under the CIEL dictionary definitions, aiming to easily integrate them into OpenMRS implementations around the worldwide community of developers interested in neglected tropical diseases.
